# Development, Molecular Docking, and Anti-Anemia Potential of Polyherbal Formulation

**DOI:** 10.3390/biology14081052

**Published:** 2025-08-14

**Authors:** Deepak Bharati, Sakshi Nirhali, Abhijeet Puri, Popat Mohite, Sudarshan Singh

**Affiliations:** 1 Department of Pharmacology, AETs St. John Institute of Pharmacy and Research, Palghar 401404, Maharashtra, India; 122sakshi2004@sjipr.edu.in; 2 Department of Pharmacognosy, AETs St. John Institute of Pharmacy and Research, Palghar 401404, Maharashtra, India; abhijeetp@sjipr.edu.in; 3 Department of Pharmaceutical Chemistry, AETs St. John Institute of Pharmacy and Research, Palghar 401404, Maharashtra, India; popatm@sjipr.edu.in; 4 Office of Research Administration, Chiang Mai University, Chiang Mai 50200, Thailand; 5 Faculty of Pharmacy, Chiang Mai University, Chiang Mai 50200, Thailand

**Keywords:** hemoglobin, antioxidant, HPTLC, in silico docking, protein, iron content, ICP-AES, FRAP

## Abstract

Anemia remains a widespread public health concern, and the search for interventions demonstrating potent anti-anemic activity is critical for reducing its impact among high-risk populations. Conventional iron therapies are associated with several complications and potential adverse effects. This study explored a polyherbal approach to develop a safer and more effective alternative treatment for anemia. A molecular docking study was initially performed to screen and evaluate alizarin, catechin, kaempferol, recesmol, rubiadin, and rutin, which are known for their antioxidant and hematinic potential. Using AutoDock Vina, these compounds were docked against the target protein (PDB ID: 6MOE) with EPE and ferrous ions as controls. Rutin demonstrated the highest binding affinity of −6.4 kcal/moL, whereas alizarin and rubiadin both followed closely with −6.3 kcal/moL, while kaempferol and ellagic acid exhibited a binding affinity of −6.2 kcal/moL. In comparison, the reference compounds tested ferrous ions, and native ligand EPE (−5.0 kcal/moL) and iron (−4.8 kcal/moL), showed mild affinities. Moreover, the tested compounds demonstrated stable binding, suggesting their potential relevance in modulating anemia-related pathways. Based on the docking results and traditional therapeutic values, a polyherbal formulation (PHF) was developed using methanolic extracts of *Trigonella foenum-graecum*, *Emblica officinalis*, *Pterocarpus marsupium*, *Withania somnifera*, *Asparagus racemosus*, *Zingiber officinale*, *Rubia cordifolia*, *Boerhavia diffusa*, and *Adhatoda vasica*. Phytochemical screening via HPTLC analysis was used to quantify the presence of gallic and ellagic acids. In addition, PHF showed significant antioxidant potential (DPPH IC_50_: 14.29 µg/mL; FRAP IC_50_: 58.57 µg/mL) and iron content (98.47 ppm) values. Furthermore, in vivo evaluation using a phenylhydrazine-induced hemolytic anemia model in Sprague Dawley rats revealed that the PHF achieved complete restoration of RBCs (6.15 ± 0.04), hemoglobin (14.82 ± 0.03 g/dL), and hematocrit (43.08 ± 0.28%) in anemic rats and improved histopathological features in the liver, spleen, and bone marrow. These results demonstrate that combined molecular and pharmacological evidence support the efficacy of PHF as a promising candidate for the management of anemia by enhancing erythropoiesis, improving iron metabolism, and reducing oxidative stress.

## Simple Summary

This study presents a polyherbal formulation (PHF) as a safer and more effective alternative treatment for anemia. Molecular docking identified rutin, alizarin, and rubiadin as potential ligands to anemia-related protein targets (PDB ID: 6MOE), showing higher binding affinities than standard iron supplements. Based on these findings, a PHF was developed using the extracts from nine medicinal plants. HPTLC confirmed the presence of gallic and ellagic acids, while the antioxidant assay (DPPH IC_50_: 14.29 µg/mL; FRAP IC_50_: 58.57 µg/mL) and high iron content (98.47 ppm) supported its therapeutic potential. In vivo studies in an anemic rat model demonstrated restoration of hematological parameters and improvement in organ histology, suggesting the efficacy of the developed PHF through erythropoiesis, iron regulation, and oxidative stress reduction.

## 1. Introduction

The World Health Organization (WHO) defines anemia as a blood disorder characterized by diminished red blood cell (RBC) count or inadequate hemoglobin (Hb) levels for meeting the body’s oxygen requirements [1]. The symptoms commonly include exhaustion, feebleness, light headedness, cephalalgia, and breathlessness. Anemia poses a considerable global health issue, affecting approximately 24.8% of individuals worldwide, with a higher prevalence among preschool-aged children and women, including those who are pregnant [2]. The WHO classifies anemia based on hemoglobin concentration, with thresholds set at 13 g/dL for males and 12 g/dL for females. The widespread nature of this condition emphasizes the critical need for focused strategies to reduce its impact, particularly among susceptible groups [3]. Anemia is managed using various therapeutic approaches, depending on the underlying cause, type, and severity of the condition. Common medical treatments include iron supplementation (oral or intravenous), vitamin B12 and folic acid supplementation, erythropoiesis-stimulating agents (ESAs), and blood transfusions in severe cases. In patients with chronic kidney disease or anemia associated with cancer [4], ESAs such as epoetin alfa and darbepoetin alfa are frequently used to stimulate red blood cell production. For hemolytic or aplastic anemia, immunosuppressive therapies, corticosteroids, or bone marrow transplantation may be required. Dietary modifications, including increased intake of iron-rich foods, are also recommended alongside pharmacological therapies to improve hemoglobin levels [5]. In recent years, there has been growing interest in the use of natural products for anemia management, particularly because of their fewer side effects and potential to provide additional health benefits. Medicinal plants are rich in iron, vitamins, flavonoids, and polyphenols [6], widely explored for their hematological properties. Herbal formulations and polyherbal extracts are being studied for their ability to enhance iron absorption, stimulate erythropoiesis, and mitigate oxidative stress, thereby offering complementary or alternative strategies for anemia therapy [7,8].

The use of oral iron supplements has several drawbacks, including poor absorption and challenges in patient compliance. High doses of these supplements can lead to severe health issues, potentially contributing to neurological disorders or increasing the risk of cancer. These concerns highlight the need to identify safe and effective alternatives for managing anemia. In recent years, herbal medicine has attracted significant worldwide attention owing to its substantial contribution to healthcare and its potential as an alternative to traditional medical treatments. Plants with medicinal properties are known to be abundant sources of bioactive phytochemicals, such as phenols, flavonoids, alkaloids, steroids, and phytates, which demonstrate various therapeutic effects across multiple bodily systems, including the nervous, respiratory, reproductive, renal, circulatory, and visual systems [9,10]. Despite their widespread use, many medicinal plants are used without proper scientific training or a thorough understanding of their safety and effectiveness. Consequently, rigorous scientific validation is crucial to confirm their therapeutic potential and ensure their safe and efficacious application in healthcare practice [11].

Recently, numerous plants from tropical and subtropical regions have attracted significant interest in Western health food markets owing to their exceptional nutritional value, particularly as rich sources of essential minerals and vitamin C. Fenugreek seeds (*Trigonella foenum-graecum*) stand out for their high iron content and proven ability to prevent the oxidation of RBCs, making them a promising natural solution for anemia prevention and treatment [12]. Research has shown that ginger rhizomes (*Zingiber officinale*) can boost RBC counts and hemoglobin (Hb) levels [13]. Vasaka (*Adhatoda vasica*) leaves have been found to significantly elevate RBCs, white blood cell (WBC), Hb, and hematocrit (HCT) levels, and the roots of punarnava (*Boerhavia diffusa*) possess antioxidant properties and are traditionally employed in anemia treatment [14]. Similarly, Manjishtha (*Rubia cordifolia*) roots are renowned for their blood-purifying effects [15]. Studies have shown that pomegranate (*Punica granatum*) peel can increase the RBC and Hb counts [16]. Ashwagandha (*Withania somnifera*) roots have also been shown to elevate RBC and Hb levels [17]. *Emblica officinalis* (amla) fruits have been found to enhance RBC, Hb, and WBC counts and are used to treat iron-deficiency anemia. Finally, shatavari (*Asparagus racemosus*) roots have been reported to increase the RBC count, Hb level, white blood cell (WBC), and platelet count. In addition to their individual hematinic effects, these plants are rich in diverse bioactive compounds, such as aliazrin, catechin, kaempferol, rubiadin, rutin [18], gallic acid (GA) [19], and ellagic acid (EA), which possess significant antioxidant, anti-inflammatory, and anti-anemic activities [20]. When used in combination, these phytochemicals from plant extracts are hypothesized to exhibit synergistic effects that enhance iron absorption, promote RBC production, and mitigate oxidative stress [21]. These mechanisms are vital for the effective treatment of anemia.

The rationale for combining these plants into a polyherbal formulation (PHF) was supported by molecular docking, which serves as a predictive in silico tool to screen and validate the interactions of the selected phytoconstituents with the target protein relevant to anemia. Molecular docking provides information on key interactions between the chosen phytoconstituents and 6MOE protein, a heme-binding protein involved in iron metabolism and erythropoiesis regulation [22]. Assuming the limitations of conventional iron therapies and the growing interest in plant-based alternatives, the development of a scientifically validated polyherbal formulation offers a promising avenue for safer and more effective anemia management. This study was designed to integrate ethnopharmacological knowledge with molecular docking approaches to rationally select and validate phytoconstituents targeting the 6MOE heme-binding protein, a key regulator of iron metabolism and erythropoiesis. Based on these findings, a polyherbal formulation (PHF) was developed using methanolic extracts of traditionally used medicinal plants and subsequently evaluated for its antioxidant activity, iron content, and in vivo anti-anemic efficacy in a phenylhydrazine-induced anemia model in rats. The study aims to provide both molecular and pharmacological evidence supporting the therapeutic potential of PHF as a novel and holistic anti-anemic intervention.

## 2. Materials and Methods

### 2.1. Animal Procurement

Healthy male Sprague Dawley rats (150 and 250 g) were procured from Lascmi Biopharms Pvt. Ltd. (Pune, India) [REG NO. 1277/PO/RcDT/S/09/CCSEA]. The animals were kept in polypropylene cages and reared in the animal house of St. John Institute of Pharmacy and Research, Palghar, at a temperature of 25 ± 2 °C, with relative humidity maintained at 50 ± 10% and a light/dark cycle of 12 h. Animals were fed ad libitum with a laboratory standard pellet diet. All studies were conducted in accordance with guidelines approved by the Institutional Animal Ethical Committee of the Institute (SJIPR/IAEC/04/23-24 dated). The study was conducted during the period from June 2023 to December 2024.

### 2.2. Chemicals and Instruments

Phenylhydrazine hydrochloride (PHZ) was purchased from LOBA Chemicals, Mumbai, India (AR Grade). 2,2-diphenyl-1-picrylhydrazyl (AR grade) was purchased from Sigma–Aldrich, Mumbai, India. Gallic acid (Yucca/GA/2022/07/18) and ellagic acid (Yucca/EA/2023/01/15) were purchased from Yucca Enterprises (Mumbai, India). Livogen XT tablet (Procter & Gamble Health Ltd., Mumbai, India) was purchased from a popular medical store in Palghar, India. A hematoanalyzer (Agappe: Mispa Count X, Kerala, India) was used for blood analysis.

### 2.3. Collection and Procurement of Plant Material

The powdered forms of the eight plant species were procured from Yucca Enterprises, located in Wadala (E), Mumbai, India. These included *Trigonella foenum-graecum*, *Emblica officinalis*, *Pterocarpus marsupium*, *Withania somnifera*, *Asparagus racemosus*, *Zingiber officinale*, *Rubia cordifolia*, and *Boerhavia diffusa.* PHF was prepared using the leaves of *Adhatoda vasica* (Vasaka) and peels of *Punica granatum* (pomegranate). Vasaka leaves were sourced from St. John Technical and Educational Campus in Palghar, Maharashtra, India (19.7070° N, 72.7851° E). The leaves were cleaned with tap water, followed by shade drying and subsequent drying in a hot air oven (Metalab, Pune, India) at the optimal temperature for two days. After vein removal, the leaves were ground and passed through a sieve (No. 30) to produce a coarse powder for extraction. Pomegranate peels were acquired from a local Palghar market in Maharashtra, India. The peels were processed into powders using an identical method [23]. Methanol (96%) (AR grade) was used as the extraction solvent.

### 2.4. Preliminary Qualitative Phytochemical Analysis

The powdered drugs were subjected to qualitative phytochemical analysis to detect the presence of essential phytochemicals that may be employed for the treatment of anemia. The powdered drugs were analyzed to confirm the presence of alkaloids, flavonoids, tannins, saponins, anthraquinone glycosides, triterpenoids, and steroidal glycosides, as previously reported [24]. The phytochemical analysis revealed the presence of alkaloids, flavonoids, tannins, saponins, anthraquinone glycosides, triterpenoids, and steroidal glycosides. Alkaloids are nitrogenous compounds with notable analgesic, antimicrobial, and anticancer activities, while flavonoids are polyphenols exhibiting strong antioxidant, anti-inflammatory, and cardioprotective effects. Tannins, known for their astringent nature, possess antimicrobial and antioxidant properties, and saponins, characterized by their foaming ability, display antifungal and cholesterol-lowering activities. Anthraquinone glycosides act as natural laxatives with additional antimicrobial and anti-inflammatory effects, whereas triterpenoids, often bitter terpenes, are recognized for their hepatoprotective, antiviral, and anticancer roles. Steroidal glycosides, potent bioactive compounds with a steroid backbone, are valued for their cardiac stimulant and anticancer properties. In one of the studies, Punica granatum showed no toxicity [25]. Rubia cordifolia contains anthraquinones, such as alizarin and rubiadin [26], and Adhatoda vasica contains vasicine, reported to show a safety profile as compared to toxicity in terms of carcinogenicity [27].

### 2.5. In Silico Molecular Docking

#### 2.5.1. Ligand Selection and Preparation

A targeted set of phytoconstituents reported in the literature was selected for molecular docking studies to explore their interactions with proteins implicated in anemia. Specifically, alizarin, catechin, kaempferol, recesmol, rubiadin, rutin, GA, and EA were chosen because of their documented antioxidant, anti-inflammatory, and hematinic properties [20]. For benchmarking purposes, the native ligand, EPE, and ferrous ions served as reference standards. Three-dimensional structures of all ligands were either obtained from the PubChem database (accessed on 8 June 2025) or constructed de novo using ChemDraw 22 or ChemSketch 12.0. Following the acquisition, these structures underwent energy minimization via the MMFF94 force field in Open Babel (version 3.1.1.)to ensure optimized conformations. The minimized structures were saved in the PDB format and subsequently converted to the PDBQT format using AutoDock Tools, (4.0) facilitating their use in docking simulations.

#### 2.5.2. Protein Preparation

The crystal structure of the target protein (PDB ID: 6MOE) was retrieved from the RCSB Protein Data Bank (PDB) (accessed on 8 June 2025). Pre-processing steps included the removal of water molecules and extraneous heteroatoms, as well as the addition of polar hydrogen atoms and Kollman charges to optimize the receptor for docking. The processed protein structure was then saved in the PDBQT format. Docking simulations were performed in AutoDock Vina (autodock_vina_1_1_2_win32.msi) with a defined grid box encompassing the active site of the protein to ensure comprehensive coverage of the binding pocket. Each ligand was individually docked into the active site, and the resulting binding affinities (reported in kcal/moL) were recorded for the most favorable binding positions [28].

#### 2.5.3. Docking Simulation and Analysis

Post-docking analyses were conducted using visualization platforms such as Discovery Studio Visualizer (2021) to assess ligand–protein interactions and confirm appropriate binding orientations. Binding affinities and root-mean-square deviation (RMSD) values were documented to evaluate the reliability and consistency of docking results [29].

### 2.6. Preparation of Polyherbal Formulation

PHF was developed using powdered crude drugs from ten medicinal plants: *Trigonella foenum-graecum*, *Emblica officinalis*, *Pterocarpus marsupium*, *Withania somnifera*, *Asparagus racemosus*, *Zingiber officinale*, *Rubia cordifolia*, *Boerhavia diffusa*, *Adhatoda vasica*, and *Punica granatum*. The raw powders of these crude drugs (Table 1) were combined and macerated in 400 mL of methanol for 24 h and 3 cycles. Subsequently, the solvent was removed using a rotary evaporator, and the extracts were dried, ground into a fine powder, and stored in a desiccator [23]. The percentage yield of PHF was found to be 4.719%. A polyherbal formulation (PHF) was prepared by combining selected medicinal plant parts, as shown in Table 1, to achieve a total dosage of 500 mg per capsule. The formulation includes seeds of *Trigonella foenum-graecum*, rhizomes of *Zingiber officinale*, and leaves of *Adhatoda vasica*, along with Pterocarpus marsupium gum and the pericarp of *Emblica officinalis*. Additional components include the peels of *Punica granatum* and roots of *Withania somnifera*, *Boerhavia diffusa*, *Asparagus racemosus*, and *Rubia cordifolia*. These ingredients were selected based on their known hematinic, antioxidant, and adaptogenic properties. All the components were accurately weighed and blended to ensure uniformity and synergistic therapeutic action (Table 1).

### 2.7. Thin-Layer Chromatography Analysis

The PHF sample (5 mg) was introduced into a test tube and dissolved in methanol (5 mL) before sonication using a sonicator (Inco, Chandigarh, India). Subsequently, the PHF samples were examined using thin-layer chromatography (TLC) on Merck silica gel 60 F254 pre-coated plates (Merck, Specialities Ltd., Mumbai, India). The samples were placed in glass capillary tubes that were sealed at one end. TLC plates were developed using the ascending elution technique, and visualization was performed in a UV cabinet, as reported [30] (Table 2).

### 2.8. High-Performance Thin-Layer Chromatography

PHF (0.25 g) was diluted with methanol (5 mL). Standard GA (10 mg) was diluted with methanol (10 mL). Standard EA (10 mg) was added to hydrochloric acid (HCl) (1 drop), diluted in methanol (10 mL), and filtered through a 0.45 μm nylon filter. The standard and test solutions were subjected to high-performance thin-layer chromatography (HPTLC) using a Camag TLC Scanner 3 ‘170308’ (HPTLC System: QCE015), Anchrom, Switzerland at GeoChem Laboratories Pvt. Ltd., Kanjur Marg, Mumbai, India. Chromatography was performed on a 10 × 10 cm HPTLC Silica gel 60 F254 plate with a mobile phase (Table 3). PHF, EA, and GA were applied as bands 8 mm wide and 14 mm apart. Chromatograms were obtained for both EA and GA. The peaks were identified by comparing the Rf values reported previously [31].

### 2.9. Estimation of Iron Content

The iron (Fe) content of the samples was determined using inductively coupled plasma–optical emission spectroscopy (ICP-AES). The analysis was performed with an ICP-AES instrument, Spectroarcos, Kleve, Germany (Model: MY17320002, Software Version: 7.3.1.9507) following the procedure reported previously [7], with slight modifications. To estimate the iron content, samples were submitted to Shraddha Analytical Services, Ghatkopar, Mumbai. For sample preparation, accurately weighed powdered plant material was subjected to acid digestion using a mixture of concentrated nitric acid (HNO_3_) and hydrochloric acid (HCl) in a ratio of 3:1 (*v*/*v*) under controlled heating until complete dissolution was achieved. The digested samples were filtered through Whatman No. 42 filter paper, and the filtrate was diluted to a known volume with deionized water.

The prepared samples were submitted to Shraddha Analytical Services, Ghatkopar, Mumbai, for ICP-AES analysis. Calibration of the instrument was carried out using certified iron standard solutions (prepared from Fe(NO_3_)_3_) at different concentrations to generate a calibration curve. The operating parameters, such as plasma gas flow rate, auxiliary gas flow, nebulizer pressure, and observation wavelength (238.204 nm for Fe), were optimized according to the manufacturer’s recommendations to ensure maximum sensitivity and accuracy. Each sample was analyzed in triplicate, and the iron content was expressed in mg of Fe per 100 g of dry weight.

### 2.10. Phytoconstituent Analysis

#### 2.10.1. Total Phenolic Content

Total phenolic content was measured as previously reported [32]. Briefly, five concentrations of GA (10 mg in 100 mL water) were prepared at concentrations of 20, 40, 60, 80, and 100 ppm. PHF (25 mg) was added to distilled water (100 mL), sonicated for 15 min, and filtered. To assess total phenolic content (TPC), 2N Folin–Ciocalteu reagent (0.5 mL) was added to the solution and mixed thoroughly using a vortex mixer. After 4 min, 1.5 mL of 0.7 M Na_2_CO_3_ solution was added, and the volume was adjusted with distilled water. The mixture was vortexed and incubated in the dark for 40 min, and absorbance was measured at 725 nm using a UV–Vis spectrophotometer (Shimadzu UV-Vis 1900, Kyoto, Japan). This procedure was performed in triplicate to ensure accuracy. TPC was calculated using a GA calibration curve and expressed as GA equivalents using the following formula:

$$TPC=\frac{C\times V}{M}$$

where T = TPC (milligram per gram of plant extract); C = Conc. of GA (milligram per milliliter); V = PHF vol. (milliliter); M = weight of PHF (gram).

#### 2.10.2. Total Flavonoid Content

Total flavonoid content was measured as previously reported [33]. Briefly, quercetin (10 mg) and PHF (25 mg) were dissolved in 100 mL of methanol to prepare quercetin (10–60 µg/mL) and PHF (40 ppm) solutions. To each solution, 0.15 mL of 1.0 moL/L NaNO_2_ was added and incubated for 3 min, followed by the addition of 0.15 mL of 10% *w*/*v* AlCl_3_ and further incubation for 3 min. Subsequently, 1 mL of NaOH was added, and the volume was adjusted to 10 mL with methanol. The mixtures were incubated in the dark for 40 min, and absorbance was measured at 510 nm using a UV–Vis spectrophotometer (Shimadzu UV-Vis 1900, Kyoto, Japan). The total flavonoid content was calculated as quercetin equivalents (QE/mL) using a quercetin calibration curve. All samples were tested in triplicate.

$$TFC=\frac{C\times V}{M}$$

where T = TFC (milligram per gram of plant extract); C = Conc. of quercetin (milligram per milliliter); V = PHF vol. (milliliter); M = weight of PHF (gram).

#### 2.10.3. Total Tannin Content

Total tannin content (TTC) was measured as previously reported [34]. Stock solutions of PHF (25 ppm) and GA (10 ppm; standard) were prepared in methanol. Serial dilutions of GA (10, 20, 30, 40, 50, and 100 µg/mL) were prepared. To each sample, 3 mL of 4% vanillin solution and 1.5 mL of 1M hydrochloric acid were added and vortexed. The volume was adjusted to 10 mL with methanol; the reaction mixtures were incubated for 5 min at room temperature; and absorbance was measured at 500 nm using a UV–Vis spectrophotometer (Shimadzu UV-Vis-1900, Kyoto, Japan). The TTC of PHF was determined from the GA calibration curve and expressed as mg GA equivalents/mL (GA/mL). All samples were tested in triplicate.

### 2.11. Antioxidant Activity

#### 2.11.1. 2,2-Diphenyl-1-Picrylhydrazyl (DPPH) Assay

Antioxidant activity was measured using 2,2-diphenyl-1-picrylhydrazyl (DPPH), as previously reported [35]. Briefly, a stock solution of DPPH was prepared by dissolving DPPH (10 mg) in methanol (100 mL) and incubating it in the dark for 30 min. For sample and standard preparation, PHF (25 mg) and ascorbic acid (AA; 10 mg) were dissolved in methanol (100 mL). Five concentrations (20, 40, 60, 80, and 100 ppm) were prepared by diluting each solution (1 mL) with DPPH stock solution (3 mL) and adjusting the volume to 10 mL with methanol. The reaction mixtures were incubated in the dark for 15 min, and absorbance was measured at 517 nm using a UV–Vis spectrophotometer (Shimadzu UV-Vis-1900, Kyoto, Japan). The percentage of inhibition and IC_50_ values of PHF and AA were calculated.

$$Inhibition(\%)=\frac{\left(AB-AA\right)}{AB}\times 100$$

where AB = absorption of blank sample and AA = absorption of the tested extract solution.

#### 2.11.2. Ferric Reducing Antioxidant Power Assay

Ferric reducing antioxidant power was measured as reported previously [36]. Briefly, a 25 ppm PHF and 10 ppm GA (standard) stock solution was prepared in methanol (100 mL). Five concentrations (0.2, 0.4, 0.6, 0.8, and 1 mg/mL) of PHF and GA were prepared. To the reaction mixture, 2 mL of phosphate buffer (0.2 M, pH 6.6) and 2 mL of 1% potassium ferricyanide (K_3_Fe(CN)_6_) were added, followed by incubation for 20 min. Subsequently, 2 mL of 10% trichloroacetic acid was added, and the mixture was centrifuged at 1000 rpm for 10 min. The supernatant (2 mL) was aspirated, combined with 2 mL of distilled water, and mixed with 1 mL of 0.1% ferric chloride (FeCl_3_). All samples were analyzed in triplicate, and absorbance was recorded at 700 nm using a UV–Vis spectrophotometer (Shimadzu UV-Vis-1900, Kyoto, Japan). The percentage inhibition and IC_50_ values for PHF and GA were determined.

$$Inhibition(\%)=\frac{\left(AB-AA\right)}{AB}\times 100$$

where AB = absorption of blank sample and AA = absorption of the tested extract solution.

### 2.12. Acute Toxicity Study

Acute oral toxicity studies were conducted according to the Organization for Economic Co-operation and Development (OECD) Guideline No. 425 [37]. PHF administered at a dose of 5000 mg/kg showed no adverse effects on the behavior or physical health of rats over 14 days. No tremors, salivation, diarrhea, skin changes, or behavioral alterations were observed, and no mortality or weight loss was observed. Therefore, the LD_50_ of PHF is anticipated to be greater than 5000 mg/kg.

### 2.13. Phenylhydrazine-Induced Anemia in Sprague Dawley Rats

#### 2.13.1. Experimental Induction of Hemolytic Anemia

Phenylhydrazine hydrochloride at a dose of 60 mg/kg was administered via intraperitoneal injection for two consecutive days to induce anemia. When RBC and Hb counts were reduced by 30%, rats were considered anemic [7].

#### 2.13.2. Experimental Design

The experimental animals were assigned to six groups, with each group comprising five rats [7]. Table 4 shows the treatment groups used for the anti-anemic evaluation of the polyherbal formulation (PHF). Six groups were included in the study. Group I served as the control and received no treatment. Group II was the anemic control, administered PHZ at 60 mg/kg to induce anemia. Group III received PHZ along with the standard treatment Livogen XT (9 mg iron/kg, twice daily). Groups IV, V, and VI were the test groups treated with PHZ and varying doses of PHF: 100 mg/kg once daily (Group IV), 200 mg/kg once daily (Group V), and 100 mg/kg twice daily (Group VI). This setup allowed for a comparative evaluation of PHF’s dose-dependent anti-anemic activity.

#### 2.13.3. Blood Withdrawal and Histopathology Study

Blood samples (0.5 mL) were collected from the retro-orbital sinus in EDTA blood collection tubes on days 0 (prior to PHZ administration), 3, 7, 10, and 15 (after PHZ administration). Hematological parameters, such as RBC count, Hb, and HCT, were analyzed using a Hematology Analyzer (Agappe: Mispa Count X). Blood samples were outsourced to the Sahyog Diagnosis Pathology Lab, Palghar, and tested as reported [7]. On day 15, the animals were euthanized, and the spleen, liver, and femur were isolated for subsequent bone marrow analysis. The collected tissues were processed, stained with hematoxylin and eosin (H&E), and examined under a light microscope to assess histopathological changes. The organs for histopathology were outsourced to UNIQUE Biodiagnostics Vet Path LAB (Parel, Mumbai, India), and sections were prepared as reported [7].

### 2.14. Statistical Analysis

Statistical analyses were conducted using the GraphPad Prism software (version 5.0) by GraphPad Software, Inc., a company based in San Diego, CA, USA. Data are presented as mean ± standard error of the mean (SEM). One-way analysis of variance (ANOVA) followed by Tukey’s post hoc test were used to determine statistical significance, with a threshold set at *p* < 0.05.

## 3. Results and Discussion

PHF, consisting of selected medicinal plants recognized for their hematological and antioxidant properties, presents a promising natural approach for the management of anemia. Each component was meticulously selected based on its traditional application and therapeutic potential in enhancing iron absorption, stimulating erythropoiesis, and supporting systemic health. The uniform blending of these botanicals ensures synergistic effects, potentially improving hemoglobin levels and addressing associated symptoms. This study underscores the importance of integrating plant-based therapies as effective and safer alternatives to conventional anemia treatments.

### 3.1. Phytochemical Screening

The phytochemical screening of plant extracts is essential to identify the main chemical constituents and bioactive compounds present, such as alkaloids, flavonoids, tannins, etc. These assessments reveal which compounds are predominant and provide a foundational step for discovering bioactive agents that may be useful in developing therapeutic drugs and alternative treatments. Thus, the phytochemical screening of the individual plant extracts and the final polyherbal formulation (PHF) revealed the presence of various bioactive constituents known for their therapeutic relevance in anemia management. The PHF tested positive for all six major classes of phytoconstituents—alkaloids, flavonoids, saponins, tannins, anthraquinone glycosides, and triterpenes/steroidal glycosides—demonstrating its phytochemical richness and synergistic potential. Alkaloids were present in *Trigonella foenum-graecum* (TFG), *Withania somnifera* (WS), *Asparagus racemosus* (AR), *Boerhavia diffusa* (BD), and *Adhatoda vasica* (AV), contributing to hematopoietic stimulation and antioxidant effects. Flavonoids, known for enhancing iron absorption and exhibiting strong antioxidant properties, were found in *Emblica officinalis* [38], *Pterocarpus marsupium* (PM), *Zingiber officinale* (ZO), *Rubia cordifolia* (RC), *Boerhavia diffusa* (BD), and *Punica granatum* (PG), and they were present in the PHF. Saponins, although present in fewer plants, such as TFG and AR, were also detected in the PHF, suggesting their role in improving iron bioavailability. Tannins were found in EO, PM, ZO, RC, BD, and PG; while excessive tannins may hinder iron absorption, their moderate presence may offer antioxidant and antimicrobial benefits. Anthraquinone glycosides were uniquely identified in Rubia cordifolia and carried over to the PHF, potentially contributing to hematopoietic and detoxifying functions. Additionally, triterpenes and steroidal glycosides were present in TFG, WS, AR, ZO, and BD, indicating potential anti-inflammatory and immunomodulatory roles. The cumulative presence of these phytochemicals in the PHF supports its therapeutic efficacy by offering a multifaceted mechanism of action involving enhanced erythropoiesis, improved iron metabolism, and oxidative stress reduction (Table 5).

### 3.2. Thin-Layer Chromatography

The TLC method was employed for further optimization, quantification, and separation. Based on the results, these three solvent systems were selected for TLC analysis. Phytochemical detection based on colorimetric spotting methods provides visual confirmation of key constituents [39]. The presence of alkaloids was indicated by the appearance of an orange spot at an Rf value of 1.2 cm, suggesting that nitrogen-containing compounds are known for their pharmacological activities, such as analgesic and antimicrobial effects. The black spots observed for flavonoids and tannins at an Rf value of 1.3 cm confirmed their presence; both are potent antioxidants and contribute to anti-inflammatory and astringent properties. The brown spots indicating saponins confirmed (Rf value −1.16 cm) their occurrence, which are known for their surfactant properties, contributing to antimicrobial and immune-boosting effects. These color-based indicators supported the qualitative assessment of phytochemicals in the studied extracts (Table 6).

### 3.3. High-Performance Thin-Layer Chromatography

**Figure 1:**
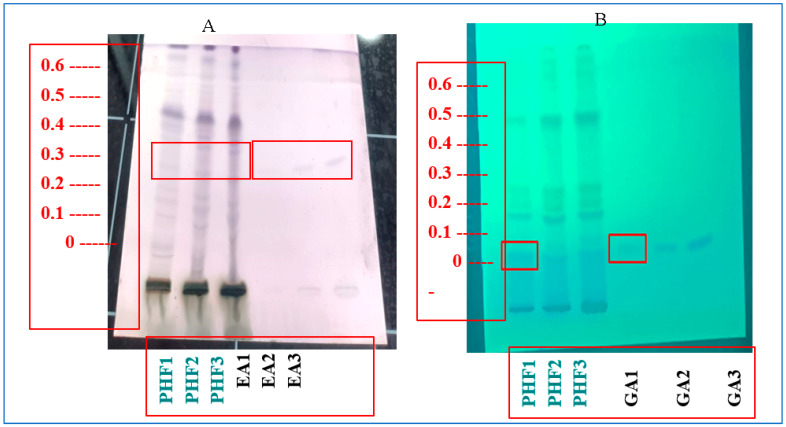
HPTLC and marker-based standardization of (**A**) EA and (**B**) GA.

HPTLC analysis effectively detected distinct peaks that matched the reference standards for GA and EA at their respective Rf values. The similarity between the peak profiles of the standards and the PHF sample, as depicted in Figure 1, verifies the presence of significant phenolic compounds of gallic and ellagic acids in the formulation. HPTLC analysis revealed that 0.25 g of PHF contained 0.18% of GA (an antioxidant that improves RBC count) [31] and 0.86% of EA (an anti-sickling agent that improves RBC and Hb count [31]). GA and EA are known for their strong antioxidant, anti-inflammatory, and therapeutic properties, which likely enhance the overall effectiveness of PHF. This qualitative verification supports the potential health benefits of the formulation and confirms the inclusion of bioactive plant constituents that contribute to its pharmacological effects [31].

### 3.4. Estimation of Iron, Phenol, Flavonoid, and Tannin Content in Polyherbal Formulation

Phytochemical screening was performed to identify the major classes of bioactive compounds present in the polyherbal formulation (PHF), such as alkaloids, flavonoids, tannins, saponins, anthraquinone glycosides, and triterpenes. This qualitative analysis is essential to scientifically validate the traditional medicinal use of the constituent plants and to understand the potential therapeutic mechanisms underlying the formulation’s anti-anemic activity. The quantitative estimation of iron content was conducted to confirm the PHF’s capacity to serve as a natural source of dietary iron, a critical element in managing iron-deficiency anemia [40]. Quantitative analysis of the PHF revealed a significant iron content of 98.47 ppm (9.847 mg/100 mL), indicating its potential to supplement dietary iron and aid in the management of iron-deficiency anemia. Additionally, the PHF demonstrated a high concentration of bioactive polyphenols, with a total phenolic content of 73.97 mg GAE/g extract, which may contribute to antioxidant and anti-inflammatory effects that support hematopoiesis and overall systemic health [41]. Furthermore, the formulation exhibited a high flavonoid content (89.32 mg quercetin/g extract), suggesting strong free radical scavenging activity, which is beneficial in protecting red blood cells from oxidative stress. The presence of tannin content (61.89 mg GAE/g extract) also adds to the formulation’s astringent and antimicrobial properties, potentially aiding gut health and iron absorption. Tannins possess well-established antioxidant, antimicrobial, and astringent properties that show overall contribution to the anti-anemic potential in the formulation. Tannin intake is known to inhibit non-heme iron absorption by forming insoluble complexes. The balanced tannin levels in this formulation, in combination with flavonoids and polyphenols, may exert a protective and supportive role in enhancing hematopoiesis and improving iron utilization [42]. Thus, the quantified tannin content contributes meaningfully to the PHF’s anti-anemic and health-promoting effects [43].

### 3.5. In Silico Molecular Docking

Molecular docking analysis was performed to evaluate the interaction potential of the selected natural compounds with a target protein involved in iron metabolism, with an emphasis on their potential anti-anemic properties. The results focused on binding affinity values (kcal/moL), where increasingly negative values suggest stronger and more stable protein–ligand interactions. Among the investigated ligands, rutin demonstrated the highest binding affinity (−6.4 kcal/moL). Alizarin and rubiadin both followed closely with −6.3 kcal/moL, while kaempferol and EA each exhibited a binding affinity of −6.2 kcal/moL. In comparison, the reference compounds, namely the native ligand EPE (−5.0 kcal/moL) and iron (−4.8 kcal/moL), showed weaker affinities. This suggests that the selected phytochemicals may interact more effectively with the target protein than the conventional controls. Rutin, which is well documented for its antioxidant and vascular-protective properties, may play a substantive role in counteracting the oxidative stress associated with iron-deficiency anemia. Its strong binding affinity indicates a potential mechanism for promoting hemoglobin synthesis and supporting erythropoiesis. Similarly, alizarin and rubiadin anthraquinones, traditionally used in Ayurvedic medicine for hematological disorders, exhibit notable binding, implying possible modulation of iron storage or transport proteins and, consequently, support of red blood cell production. Catechin and kaempferol, both recognized for their antioxidant and iron-chelating capacities, also demonstrated favorable binding. This finding supports the hypothesis that they may mitigate oxidative damage and enhance iron bioavailability. While gallic acid showed a comparatively lower binding affinity (−4.6 kcal/moL), EA maintained a robust interaction profile. In addition to its antioxidant properties, EA can contribute to the maintenance of red blood cell integrity and longevity. Therefore, the superior binding affinities for these natural ligands, as compared to the reference compounds, reinforce their potential as bioactive agents in anemia management. These computational findings provide preliminary evidence to support the use of these compounds in polyherbal formulations. Such formulations may act through multiple mechanisms, including antioxidant effects, modulation of iron-regulatory proteins, and stimulation of erythropoietin pathways, thereby offering a potentially safer and more effective alternative to conventional iron supplementation. Further in vitro and in vivo studies are necessary to substantiate these computational insights and establish the therapeutic relevance of these compounds in the treatment of anemia (Table 7 and Table 8). Further biological validation through in vitro and in vivo studies is essential to substantiate these in silico results.

### 3.6. Phytochemical Screening

#### 3.6.1. 2,2-Diphenyl-1-Picrylhydrazyl (DPPH) Assay

**Figure 2:**
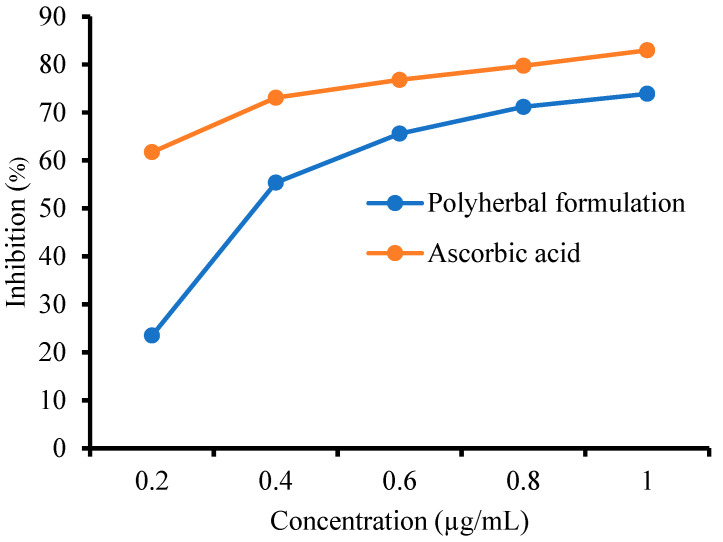
Percentage inhibition of 2,2-diphenyl-1-picrylhydrazyl by polyherbal formulation.

The scavenging activity of PHF against DPPH radicals was determined, and the results are shown in Figure 2. Ascorbic acid showed greater antioxidant potential compared to PHF. The inhibition (%) of PHF (1 µg/mL) was found to be 73.90 ± 0.32, while that of AA (1 µg/mL) was found to be 82.96 ± 0.32. The IC_50_ values of PHF and ascorbic acid were 14.2986 and 11.9199 μg/mL, respectively. When DPPH interacts with an antioxidant compound capable of donating a hydrogen atom, it undergoes reduction. This reaction results in a color change from deep violet to light yellow, indicating antioxidant activity of the compound. Thus, the greater the inhibition (%), the greater the scavenging activity [44]. Therefore, PHF showed significant scavenging of free radicals compared with AA.

#### 3.6.2. FRAP Assay

**Figure 3:**
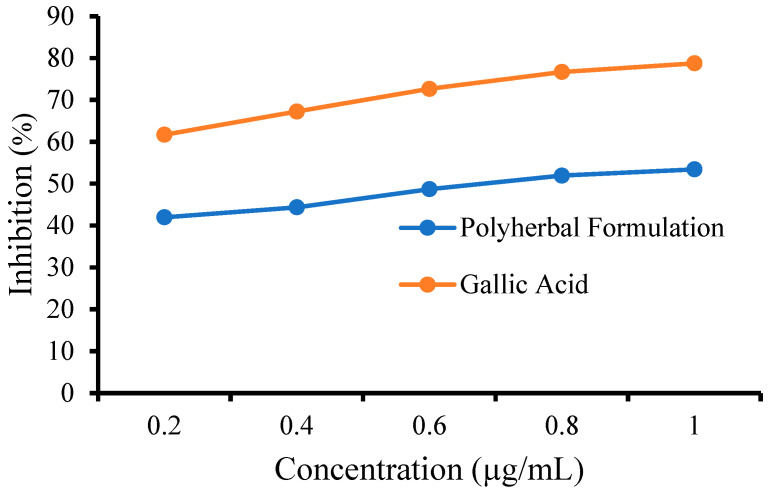
Percentage inhibition (%) of FRAP by PHF.

The FRAP assay results demonstrate that the polyherbal formulation (PHF) exhibits substantial reducing potential, confirming its antioxidant capacity. The reducing activity of PHF against the FRAP assay was determined, and the results are shown in Figure 3. GA showed greater antioxidant potential than the tested PHF. PHF (1 µg/mL) caused a reduction of ferric 2,4,6-tripyridyl-s-triazine ([Fe^3+^(TPTZ)_2_]^3+^) complex to ([Fe^2+^(TPTZ)_2_]^3+^) by 70.90 ± 0.32 compared to GA (1 µg/mL), which caused a reduction by 87.96 ± 0.32. The IC_50_ value of PHF was found to be 58.57 μg/mL and that of GA was found to be 49.99 μg/mL, respectively. The reducing agents within PHF reduce the ferricyanide (Fe^3+^) complex to its ferrous (Fe^2+^) form. Consequently, this reduction process changes the color of the test solution from yellow to green, indicating the antioxidant capacity of the sample. Thus, PHF significantly reduced the [Fe^3+^(TPTZ)_2_]^3+^ complex compared with GA, indicating its antioxidant potential [45]. These findings suggest that PHF can effectively scavenge free radicals and may offer protective effects against oxidative-stress-mediated cellular damage, which is particularly relevant in the context of anemia and related pathophysiological conditions.

### 3.7. Phenylhydrazine-Induced Anemia in Sprague Dawley Rats

The present study investigated the hematological effects of PHZ-induced anemia in Sprague Dawley rats and evaluated the therapeutic potential of PHF at various doses in comparison with a standard iron supplement (Livogen XT) [7,46]. Phenylhydrazine administration significantly reduced the RBC count, Hb levels, and HCT percentages in the anemic group, particularly on day 3 (*p* < 0.001), compared to the control group. This confirms the oxidative hemolytic nature of PHZ, which disrupts erythrocyte integrity and function. The standard group treated with Livogen XT showed a gradual and significant improvement in all parameters from day 7 onwards, indicating effective hematinic action. Among the test groups, Test Group III (100 mg/kg, twice daily) demonstrated the most notable improvement, closely approaching or matching the standard group by day 15 across all parameters (RBC, Hb, and HCT). This suggests a dose-dependent effect of PHF in the promotion of hematopoietic recovery. Test Group I (100 mg/kg, once daily) and Test Group II (200 mg/kg, once daily) also showed significant recovery compared to the anemic control, although their effects were slightly less consistent or delayed compared to those of Test Group III and the standard group. PHF exhibited a protective and restorative effect against PHZ-induced anemia, with the 100 mg/kg twice-daily dose showing the most promising hematological recovery, likely due to oxidative stress, leading to hemoglobin denaturation, membrane phospholipid degradation, and disruption of energy metabolism enzymes. The study demonstrated that anemia was successfully induced, as shown by significant reductions in RBC count, hemoglobin, and hematocrit levels in the anemic group by day 3. Among the treatment groups, Test Group III (100 mg/kg, twice daily) showed the most promising results, with steady improvement in all hematological parameters. By day 15, it achieved near-complete restoration of RBCs (6.15 ± 0.04), hemoglobin (14.82 ± 0.03 g/dL), and hematocrit (43.08 ± 0.28%), comparable to or better than the standard Livogen XT group. Test Group II (200 mg/kg, once daily) also showed strong recovery, while Test Group I had moderate effects. These findings suggest that higher or divided dosing in Test Group III enhances efficacy and may offer a potent alternative anti-anemic therapy (Table 9).

### 3.8. Histopathology Analysis

**Figure 4:**
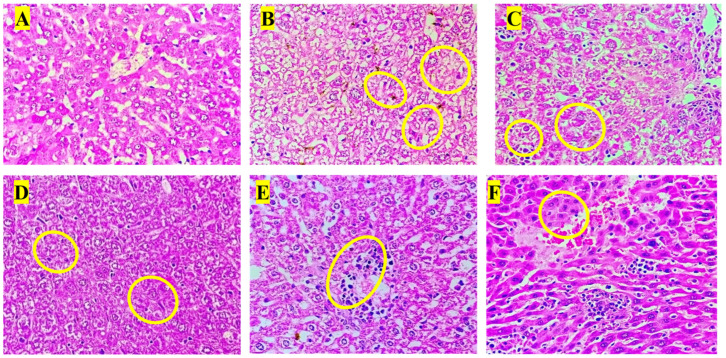
Liver sections of animals in the (**A**) normal control group, (**B**) anemic control group, (**C**) standard group, (**D**) Test Group I, (**E**) Test Group II, and (**F**) Test Group III.

Histopathological examination of liver sections revealed distinct morphological changes among the experimental groups. The normal control group (A) exhibited normal hepatic architecture with intact hepatocyte cords, clearly visible nuclei, and well-defined central veins, indicating healthy liver histology. In contrast, the anemic control group (B) showed marked pathological alterations, including hepatocellular degeneration, cytoplasmic vacuolation, and focal necrosis (yellow circles), along with sinusoidal dilatation signs consistent with phenylhydrazine-induced hemolytic anemia [47]. The standard treatment group (C) demonstrated partial restoration of hepatic structure, with reduced vacuolation and improved hepatocyte organization compared to the anemic control, although mild inflammatory infiltrates persisted. Test Group I (D) exhibited notable hepatoprotective effects, showing more organized hepatocyte arrangement and decreased necrotic regions, although occasional inflammatory cell foci remained. Test Group II (E) showed further histological improvement, characterized by reduced inflammatory infiltration and restored sinusoidal pattern, suggesting enhanced recovery. Test Group III (F) presented the most prominent hepatoprotective response, with nearly normal hepatic cord arrangement, minimal inflammatory infiltration, and absence of overt necrosis. These findings correlate with the biochemical results, indicating that the polyherbal formulation, particularly at higher doses, ameliorated anemia-induced hepatic injury by restoring cellular integrity and reducing oxidative and inflammatory damage (Figure 4).

**Figure 5:**
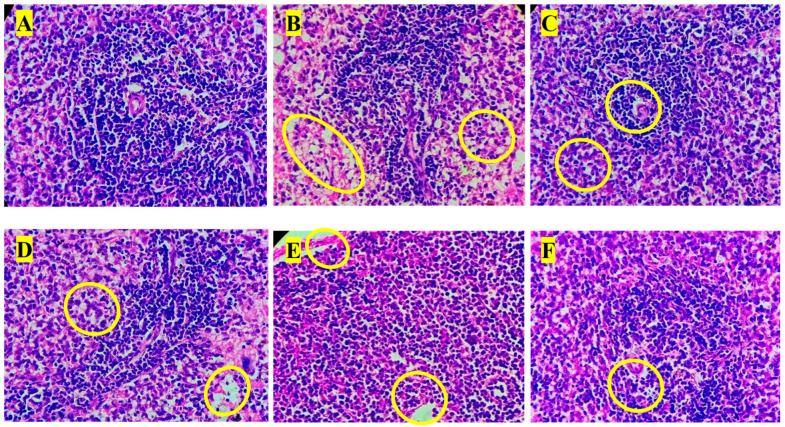
Spleen sections of animals in the (**A**) normal control group, (**B**) anemic control group, (**C**) standard group, (**D**) Test Group I, (**E**) Test Group II, and (**F**) Test Group III.

**Figure 6:**
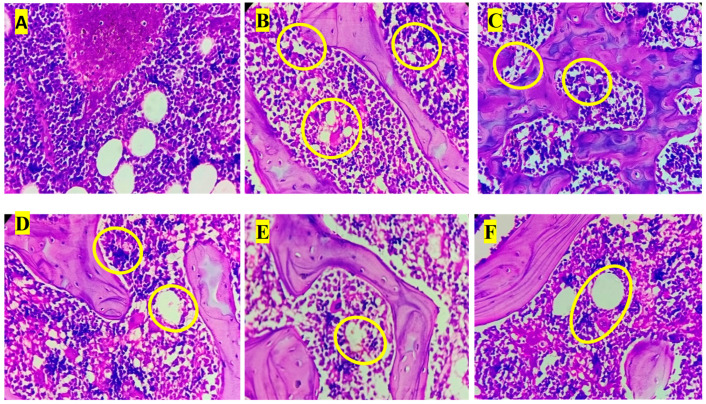
Bone marrow sections of animals in the (**A**) normal control group, (**B**) anemic control group, (**C**) standard group, (**D**) Test Group I, (**E**) Test Group II, and (**F**) Test Group III.

Histopathological observations of the liver, spleen, and bone marrow tissues revealed marked differences between the experimental groups [48]. The control group showed no abnormalities in any of the examined organs, indicating baseline healthy tissue morphology. In contrast, the anemic group exhibited the most pronounced pathological changes, including moderate-to-marked vesicular degeneration of hepatocytes, mild hemosiderosis in the liver, and moderate cellular depopulation in both the spleen (red and white pulp) and bone marrow, suggesting a significant systemic impact of anemia. In the standard group, mild vesicular degeneration of hepatocytes and mild multifocal hematopoiesis were observed in the liver, whereas the spleen and bone marrow remained unaffected, indicating a mild hepatic response with preservation of hematopoietic tissues. Test Group I showed moderate hepatic degeneration, similar to that in the anemic group, and a comparable degree of cellular depopulation in both the spleen and bone marrow, suggesting a partial but notable pathological effect. Test Group II exhibited milder changes, with the liver showing mild-to-moderate vesicular degeneration and minimal hematopoiesis, while the spleen and bone marrow demonstrated mild cellular depopulation. This indicates a reduction in severity compared to that in Test Group I and the anemia group. Finally, Test Group III displayed the least severe alterations among the test groups, with only mild hepatic degeneration and hematopoiesis, minimal spleen involvement, and minimal bone marrow depopulation, suggesting a comparatively protective or less damaging profile. Overall, the data suggest a gradient of pathological impact, with the anemic group most affected and Test Group III showing the mildest changes among the treated groups. Histopathological analysis of the liver, spleen, and bone marrow revealed moderate-grade pathological changes due to PHZ-induced anemia. Treatment with a PHF reversed these changes, restoring the tissue structure to near normal [49]. This suggests that PHF has strong anemia-treating properties and effectively mitigates anemia-induced tissue damage (Figure 5 and Figure 6).

## 4. Conclusions

The present study integrates computational, phytochemical, and in vivo evidence to demonstrate the anti-anemic potential of a polyherbal formulation (PHF) developed from traditionally used medicinal plants. Molecular docking revealed strong binding affinities, with rutin (−6.4 kcal/mol), alizarin (−6.3 kcal/mol), and rubiadin (−6.3 kcal/mol) outperforming, compared to standard ferrous ions (−4.8 kcal/mol). Phytochemical analysis confirmed the presence of gallic and ellagic acids, while antioxidant assays showed potent activity (DPPH IC_50_: 14.29 µg/mL; FRAP IC_50_: 58.57 µg/mL) and appreciable iron content (98.47 ppm). In vivo evaluation in a phenylhydrazine-induced anemia model demonstrated significant recovery of hematological parameters, including hemoglobin (from 7.2 ± 0.4 g/dL to 12.8 ± 0.5 g/dL), RBC count (from 3.1 ± 0.3 × 10^6^/µL to 6.4 ± 0.2 × 10^6^/µL), and hematocrit (from 21.5 ± 1.2% to 38.4 ± 1.5%). Histopathological analysis revealed marked restoration of the liver, spleen, and bone marrow architecture. These findings support PHF as a promising, safer, and more effective alternative to conventional iron therapy, warranting further clinical investigation to confirm its potential in human anemia management. However, to fully establish its clinical efficacy and safety, further detailed in vitro and in vivo studies are essential, particularly to validate the anti-anemic role of its flavonoid constituents.

## Figures and Tables

**Table 1:** Composition of polyherbal formulation.

Synonyms	Biological Names	Part	Quantity Sufficient up to 500 mg
Fenugreek	*Trigonella foenum-graecum*	Seeds	50 mg
Ginger	*Zingiber officinale*	Rhizomes	10 mg
Vasaka	*Adhatoda vasica*	Leaves	50 mg
Malabar Kino	*Pterocarpus marsupium*	Gum	100 mg
Amla	*Emblica officinalis*	Fruit pericarp	50 mg
Pomegranate	*Punica granatum*	Peels	20 mg
Ashwagandha	*Withania somnifera*	Roots	50 mg
Punarnava	*Boerhavia diffusa*	Roots	20 mg
Shatavari	*Asparagus racemosus*	Roots	50 mg
Manjishtha	*Rubia cordifolia*	Roots	50 mg

**Table 2:** Thin-layer chromatography analysis for alkaloids, flavonoids, tannins, and saponins.

Phytochemical Constituent	Solvent System	Visualization
Alkaloids	Dioxane: Ammonia (90:10)	Dragendroff reagent UV light at 254 nm
Flavonoids and Tannins	Chloroform: Ethyl Acetate: Formic Acid: Methanol (4:5.2:0.6:0.2)	Anisaldehyde sulphuric acid reagent and heat up to 110 °C UV light at 254 nm
Saponins	Toluene: Ethyl Acetate: Formic Acid (5:3.5:0.5)	UV light at 254 nm

**Table 3:** High-performance thin-layer chromatography for gallic acid and ellagic acid.

Active Constituent	Solvent System	Application Volume		Visualization	Derivatization
		std	phf		
Gallic acid	Toluene: Ethyl acetate: Formic acid (5:3.5:0.5)	1 μL	10 μL	280 nm	NA
Ellagic acid	Toluene: Ethyl acetate: Formic acid (5:3.5:0.5)	5 μL	5 μL	540 nm	Anisaldehyde reagent

**Table 4:** Treatment groups for anti-anemic evaluation.

Group	Treatment
Group I—Normal control	No treatment was given
Group II—Anemic control	Only PHZ 60 mg/kg
Group III—Standard group	PHZ with standard: Livogen XT tablets (9 mg of iron/kg, twice daily, p.o.)
Group IV—Test group I	PHZ with PHF 100 mg/kg, once daily, p.o.
Group V—Test group II	PHZ with PHF 200 mg/kg, once daily, p.o.
Group VI—Test group III	PHZ with PHF 100 mg/kg, twice daily, p.o.

**Table 5:** Preliminary phytochemical screening.

Phytochemical Constituents	TFG	EO	PM	WS	AR	ZO	RC	BD	AV	PG	PHF
Alkaloids	+	-	-	+	+	-	-	+	+	-	+
Flavonoids	+	+	+	-	-	+	+	+	-	+	+
Saponins	+	-	-	-	+	-	-	-	-	-	+
Tannins	+	+	+	-	-	+	+	+	-	+	+
Anthraquinone glycosides	-	-	-	-	-	-	+	-	-	-	+
Triterpenes and steroidal glycosides	+	-	-	+	+	+	-	+	-	-	+

‘+’ = Presence; ‘-’ = Absence. TFG = Trigonella foenum-graecum, EO = Emblica officinalis, PM = Pterocarpus marsupium, WS = Withania somnifera, AR = Asparagus racemosus, ZO = Zingiber officinale, RC = Rubia cordifolia, BD = Boerhavia diffusa, AV = Adhatoda vasica, PG = Punica granatum.

**Table 6:** Thin-layer chromatography of polyherbal formulation.

TLC of Alkaloids	TLC of Flavonoids and Tannins	TLC of Saponins
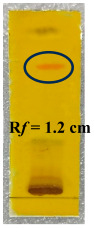	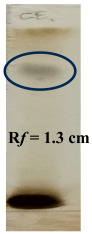	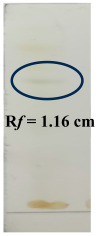

**Table 7:** Molecular docking score of the interaction between ligands and the target protein.

Ligand	Ligand Type	Binding Affinity (kcal/moL)
Alizarin	Test ligand	−6.3
Catechin	Test ligand	−6.0
Kaempferol	Test ligand	−6.2
Recesmol	Test ligand	−5.2
Rubiadin	Test ligand	−6.3
Rutin	Test ligand	−6.4
Gallic acid	Test ligand	−4.6
Ellagic acid	Test ligand	−6.2
EPE ligand	Native ligand	−5
Fe	Control	−4.8

**Table 8:** Two- and three-dimensional visualizations of molecular docking between ligands and the target protein (3MOE).

Comp.	3D Visualization	2D Visualization
**Alizarin**	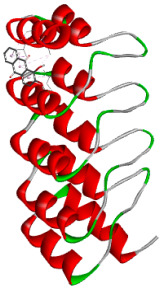	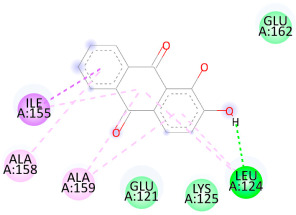
**Catechin**	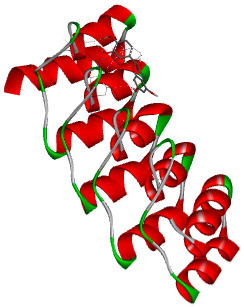	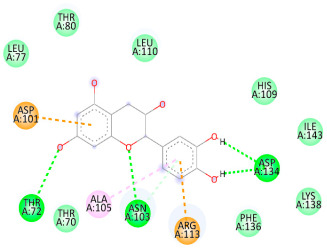
**Kaempferol**	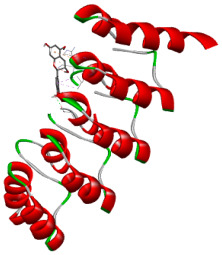	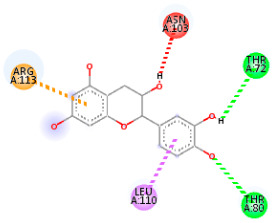
**Recesmol**	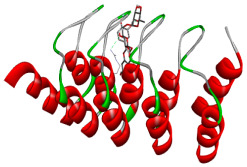	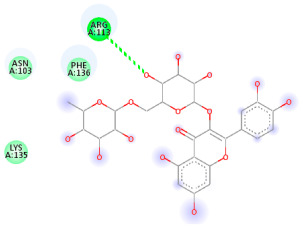
**Rubiadin**	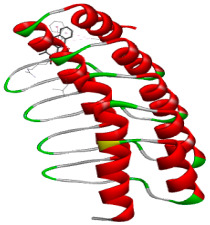	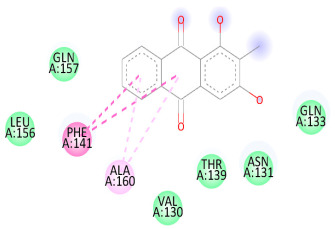
**Rutin**	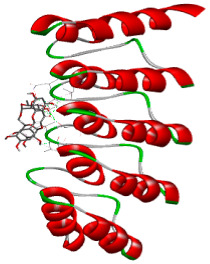	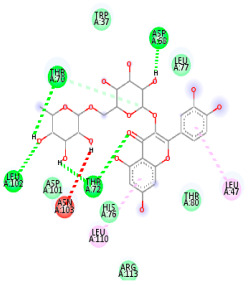
**Gallic acid**	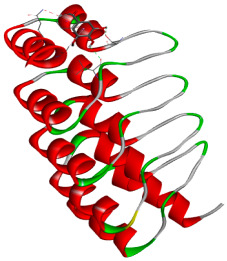	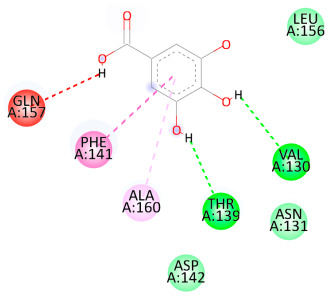
**Ellagic acid**	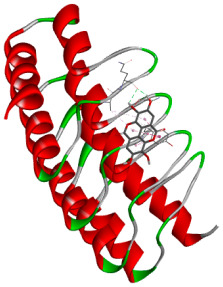	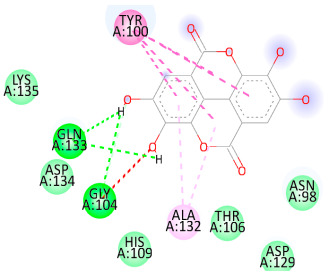
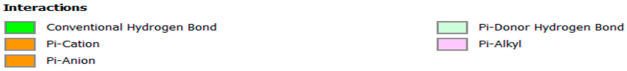		

**Table 9:** Effect of PHF on RBCs, Hb, and HCT in anemic rats.

Days	Control Group	Anemic Group	Standard Group— Livogen XT Tablet (9 mg iron/kg, Twice Daily)	Test Group I (100 mg/kg, Once Daily)	Test Group II (200 mg/kg, Once Daily)	Test Group III (100 mg/kg, Twice Daily)
**RBCs Count (106/μL)**						
Day 0	7.25 ± 0.25	6.89 ± 0.24	6.83 ± 0.01	6.75 ± 0.01	6.79 ± 0.02	6.78 ± 0.02
Day 3	7.01 ± 0.26	2.28 ± 0.28 ###	2.22 ± 0.13 ***	3.06 ± 0.13 ***	2.55 ± 0.06 ***	0.91 ± 0.04
Day 7	7.63 ± 0.21	3.59 ± 0.22 ###	5.19 ± 0.23 ***	3.95 ± 0.03 *	3.03 ± 0.05 ***	4.53 ± 0.08 ***
Day 10	7.47 ± 0.12	5.06 ± 0.17 ###	6.16 ± 0.05 ***	5.29 ± 0.03 ***	4.51 ± 0.05 ***	5.62 ± 0.17 *
Day 15	6.99 ± 0.29	6.09 ± 0.28 ##	6.53 ± 0.04 ***	4.79 ± 0.04 **	6.29 ± 0.07 ***	6.15 ± 0.04 ***
**Hemoglobin (g/dL)**						
Day 0	14.52 ± 0.41	14.07 ± 0.81	13.83 ± 0.46	14.28 ± 0.26	14.21 ± 0.25	14.28 ± 0.22
Day 3	14.22 ± 0.23	4.07 ± 0.11 ###	4.29 ± 0.06	4.27 ± 0.01	4.35 ± 0.10	3.83 ± 0.22
Day 7	12.14 ± 0.22	8.95 ± 0.17 ###	9.99 ± 0.19 ***	10.18 ± 0.04 ***	10.08 ± 0.04 ***	11.03 ± 0.22 ***
Day 10	15.1 ± 0.29	10.11 ± 0.31 ###	11.60 ± 0.02 ***	12.49 ± 0.04 ***	12.51 ± 0.04 ***	12.08 ± 0.24 ***
Day 15	14.36 ± 0.32	10.67 ± 0.35 ###	14.61 ± 0.03 ***	14.10 ± 0.03 *	14.71 ± 0.03 ***	14.82 ± 0.03 ***
**Hematocrit Count (%)**						
Day 0	43.07 ± 0.23	44.80 ± 0.18	33.17 ± 0.93	35.48 ± 1.19	33.43 ± 0.61	36.35 ± 0.13
Day 3	44.33 ± 0.31	22.28 ± 0.34 ###	21.97 ± 0.28	22.60 ± 0.28	21.73 ± 0.04	8.97 ± 0.14 ***
Day 7	42.09 ± 0.29	27.66 ± 0.59 ###	23.73 ± 0.04 ***	32.01 ± 0.99 ***	35.32 ± 0.05 ***	36.83 ± 0.20 ***
Day 10	40.34 ± 0.48	30.98 ± 0.45 ###	44.70 ± 0.21 ***	36.49 ± 0.28 ***	38.60 ± 0.60 ***	42.13 ± 0.24 ***
Day 15	41.55 ± 0.45	36.03 ± 0.67 ###	44.98 ± 0.50 ***	38.53 ± 0.34 **	41.07 ± 0.34 ***	43.08 ± 0.28 ***

Values are expressed as mean ± SEM, n = 6, * *p* < 0.05, ** *p* < 0.01, *** *p* < 0.001, ## *p* < 0.01, ### *p* < 0.001, # vs. normal control, * vs. anemia control, SEM = standard error of the mean.

## Data Availability

Data can be made available upon reasonable request to the corresponding authors.

## Abbreviations

PHF—Polyherbal Formulation, TLC—Thin-Layer Chromatography, HPTLC—High-Performance Thin-Layer Chromatography, TPC—Total Phenolic Content, TFC—Total Flavonoid Content, TTC—Total Tannin Content, DPPH—2,2-Diphenyl-1-Picrylhydrazyl, FRAP—Ferric Reducing Antioxidant Power, RBC—Red Blood Cell, Hb—Hemoglobin, HCT—Hematocrit, WHO—World Health Organization, WBC—White Blood Cell, GA—Gallic Acid, EA—Ellagic Acid, ICP-AES—Inductively Coupled Plasma–Optical Emission Spectroscopy, AA—Ascorbic Acid, HCl—Hydrochloric Acid, Na_2_CO_3_—Sodium Carbonate, NaNO_2_—Sodium Nitrite, AlCl_3_—Aluminum Chloride, NaOH—Sodium Hydroxide, K_3_Fe(CN)_6_—Potassium Ferricyanide, TCA—Trichloroacetic Acid, FeCl_3_—Ferric Chloride, OECD—Organization for Economic Co-operation and Development, PHZ—Phenylhydrazine hydrochloride, p.o.—Per Os (oral administration), IAEC—Institutional Animal Ethics Committee, CCSEA—Committee for Control and Supervision of Experiments on Animals, SEM—Standard Error of the Mean, ANOVA—Analysis of Variance.
